# Evaluation d’un enseignement par les étudiants appliquée au module « aide à la rédaction de thèse»: destiné aux internes des hôpitaux des Armées au Centre d’épidémiologie et de Santé Publique des Armées, Marseille

**DOI:** 10.11604/pamj.2016.24.236.8658

**Published:** 2016-07-14

**Authors:** Jalal Kasouati, Guillaume Velut, Xavier Deparis, Farida Touloune

**Affiliations:** 1 Faculté de Médecine et de Pharmacie Université Mohammed V de Rabat, Maroc; 2 Centre d’Epidémiologie et de Santé Publique des armées, Unité Mixte de Recherches 912 SESSTIM, Marseille, France; 3 Faculté de Médecine et de Pharmacie Université Mohammed V de Rabat, MarocX

**Keywords:** Evaluation formative, évaluation d un enseignement par les étudiants, EEE, Formative Assessment, Assessment of teaching by students, ATS

## Abstract

**Introduction:**

En éducation, l'évaluation concerne les institutions de formation, les programmes, les enseignants ou les étudiants. Elle peut être prédictive, sommative ou formative. L'Evaluation d'un Enseignement par les Etudiants (EEE) est l'un des outils de ce dernier type.

**Objectif:**

Évaluer l'enseignement de la première partie de la formation « aide à la rédaction de thèse ».

**Méthodes:**

C'est une étude transversale classée dans le cadre « EEE » qui a porté sur le module « aide à la rédaction de thèse » dispensée au CESPA au profit de 27 participants préparant leur projet de fin d'étude.

**Résultats:**

Le taux de réponse des participants présents était de 100%. D'un sexe ratio F/M de 2 et de une moyenne d'âge de 25,5 ans +/- 2,7 ans, les questionnés étaient majoritairement des internes de médecine générale. Plus de 85% affirmait n'avoir bénéficié d'aucune formation au préalable dans les domaines traités. Les participants avaient des attentes qui rejoignaient sommairement les objectifs de la formation. A part le rythme jugé non adapté par plus de la moitié, 80% des participants ont été satisfaits des autres aspects évalués et 95,8% parmi eux prévoyaient d'assister à la deuxième partie de la formation Pour tout les participants, la formation a permis d'amélioré de façon significative leur connaissance et aurais certainement un impact positif sur leurs travaux de recherche.

**Conclusion:**

Notre travail était un moyen d'installer une certaine complicité entre les étudiants et les enseignants pour atteindre un objectif commun: « AMELIORER LA FORMATION ».

## Abstract (en)

**Introduction:**

Educational assessment focuses on training institutions, programs, teachers or students. It may be predictive, summative or formative. Assessment of teaching by students (ATS) is one of the assessment tools included in the last category.

**Objective:**

Assessing teaching of the first part of the “Help thesis writing” training course.

**Material and methods:**

This is a cross-sectional study classified under the “ATS” agreement and focused on the “Help thesis writing” module provided to 27 participants preparing their end-of-study at CESPA.

**Results:**

Participant representativeness was 100%. F/M sex ratio was 2, the average age was 25.5 years +/- 2.7 years, the respondents were mainly students serving as interns in general medicine. Over 85% of students said he had received no prior training in the treated areas. The participants had expectations that roughly met the objectives of the training. Apart from teaching rhythm which was deemed unsuitable by more than half of participants, 80% of participants were satisfied with the other aspects evaluated and 95.8% of them were planning to attend the second part of training. All the participants judged the training as helpful to significantly improve their knowledge and were certain it might have a positive impact on their research projects.

**Conclusion:**

Our study aimed to develop teacher-student complicity in order to achieve a common goal: “IMPROVING THE FORMATION”.

## Introduction

Étymologiquement, le terme évaluation signifie: « déterminer une quantité ou une valeur ou l´importance de quelque chose sans recourir à une mesure directe », (ex: un rassemblement évalué à trente mille participants). Après consultation de plusieurs dictionnaires, le verbe « évaluer » peut-être défini de la façon suivante: porter un jugement sur la valeur, faire estime, donner une valeur, apprécier, donner un prix [1–4]. En éducation, le processus d'évaluation peut concerner schématiquement les établissements et les institutions de formation, les programmes et les dispositifs de formation, les enseignants ou les étudiants. Quoi qu'il en soit, l'objectif ultime sera le même; tout le monde sera d'accord avec Charles Hadji [5] pour dire: « évaluer pour mieux faire évoluer ». Ou avec Michel Crozier qui suggère: « l´évaluation n´est pas une fin en soi, mais elle doit être prise en compte par les intéressés pour améliorer leurs performances ». Dans cette optique, l'étape de l'évaluation s'avère une phase incontournable pour mettre en œuvre une formation en bonne et due forme. Une revue de l'histoire de l'évaluation des formations et des enseignements dans les universités françaises, amène à considérer 1990 comme date initiatrice. Cette date n'est en réalité que la date de publication du rapport produit par le groupe de travail piloté par Michel Crozier. Le rapport était une réponse à la question posée par Lionel Jospin, ministre de l'éducation nationale: « comment évaluer les performances pédagogiques des universités? » [6]. Depuis 1992, la question de l'évaluation des enseignements à pris un aspect formel et cela à travers des arrêtés qui se sont succédés à des intervalles plus ou moins réguliers et dans des contextes différents; notamment le contexte de la reforme Licence Master Doctorat « LMD » de 2002-2003 et celui de la démarche qualité qui prend de plus en plus de l'ampleur depuis 2006. L'évaluation d'un enseignement par les étudiants (EEE) est l'un des outils de l'évaluation formative. L'utilisation de cet outil n'est pas récente; on trouve ses premières traces dans les années 20 du XXème siècle au niveau de l'université de Harvard. C'est à partir des années 60 que l'EEE a pris plus d'importance au niveau des pays nord-américains [6–8]. Ainsi 29% des universités américaines la pratiquaient en 1973, 68% en 1983, 86% en 1993 et plus de 90% dans les années 2000 [9–13]. En France et d'après Alain Menand* « depuis 2006, les universités sont incitées à procéder à leur auto-évaluation, dont fait partie l´EEE. Mais sa mise en place est encore trop timide et très contrastée à cause de la forte réticence du corps enseignant qui craint que cette évaluation soit utilisée comme un outil de contrôle sur son travail. De plus les équipes de direction des universités, fortement mobilisées pour le passage à l´autonomie et les investissements d´avenir, n'ont souvent pas accordé la priorité à l'évaluation formative. Du coup il s´agit souvent d´initiatives locales qui se déroulent seulement dans un Département ou dans quelques formations » [14]. Objectif de l'étude: évaluer l'enseignement de la première partie de la formation « aide à la rédaction de thèse » afin de proposer une amélioration réaliste qui prenne en considération objectifs, contenu, attentes et contraintes. * Sociologue français membre de l´Académie des sciences morales et politiques **Directeur de la section des formations et diplômes de l´Agence de l'évaluation et de la recherche de l'enseignement supérieur -Aeres- en France « Avril 2007-avril 2011 »

## Méthodes

Notre étude est une étude transversale classée dans le cadre d'Evaluation d'un Enseignement par les Etudiant communément dite « EEE » qui a porté sur le module « aide à la rédaction de thèse » dispensée au CESPA sur 3 jours, du 30 Janvier au 1er Février 2013, au profit de 25 médecins et 2 nutritionnistes préparant leur thèse ou leur mémoire de fin d'étude. La formation s'est déroulée selon le programme suivant: J1 Matin: introduction à la recherche clinique; J1 Après-midi: questionnaire de recueil de données; J2 journée de formation au logiciel Epi-Info: création d'un masque de saisie et saisie des données; J3 matin: Recherche bibliographique et gestion des références (théorie); J3 après-midi: recherche bibliographique et gestion des références (TD) Les données ont été recueillies en utilisant cinq auto-questionnaires anonymes: le premier a été distribué à J1 matin au début de la formation. Il était composé de deux parties: une dédiée à l'identité du candidat et l'autre à l'évaluation de ses pré-requis et de ses attentes (annexe 1); le deuxième, le troisième et le quatrième questionnaire ont été distribués à la fin de chaque journée de cours (J1, J2 et J3). Ils comprenaient trois parties: la première pour recueillir le jugement des participants sur l'amélioration de leur connaissance dans les thèmes traités, la deuxième pour savoir s'ils jugeaient que cela leur servirait dans leurs travaux de recherche et la troisième partie avec des questions à réponses ouvertes pour recueillir le maximum de commentaires sur les points positifs, les points négatifs et les suggestions (annexes 2, 3 et 4); le dernier questionnaire a été distribué à la fin du module (J3) dans le but d'évaluer la formation dans sa totalité en focalisant sur trois axes: le contenu, le déroulement et les présentations, avec une partie dédiée aux questions à réponses ouvertes pour recueillir le maximum de commentaires sur les points positifs, les points négatifs et les suggestions. Les questionnaires ont été élaborés en s'inspirant de la littérature et en concertation avec les enseignants qui ont assuré l'animation de cette formation. La saisie et analyse statistique des données a été faite en utilisant le logiciel Epi info version 3.5.3. L'analyse bivariée a été réalisée en utilisant le test t de Student après transformation des réponses aux questions de jugement des participants en variable quantitative avec un score de 0 à 3 (0=Pas du tout d'accord, 1= Plutôt pas d'accord, 2= Plutôt d'accord, 3= Totalement d'accord), en respectant les règles d'application de chaque test et en fixant le seuil de signification à 0,05.

## Résultats

Les étudiants inscrits à cette formation étaient au nombre de 27 avec un taux de présence de 84% et un taux de réponse de 100. Seules les réponses des présents ont été prises en compte.

**Résultat du premier questionnaire identité:** d'un sexe ratio F/M de 2 et de moyenne d'âge de 25,5 ans +/- 2,7 ans, les participants étaient majoritairement des internes de médecine générale de quatre hôpitaux militaires dont 54% de l'Hôpital Sainte-Anne à Toulon (Tableau 1).

**Tableau 1:** Statut des participants

Statut	n (%)
Internes en Médecine générale	17(71)
Internes d’autre Spécialités	5(21)
Diététiciens	2(8)
**Hôpital de rattachement**	
Sainte-Anne (Toulon)	13(54)
Laveran (Marseille)	9(38)
Percy (Paris)	1(4)
Val-de-Grâce (Paris)	1(4)

**Figure 1:**
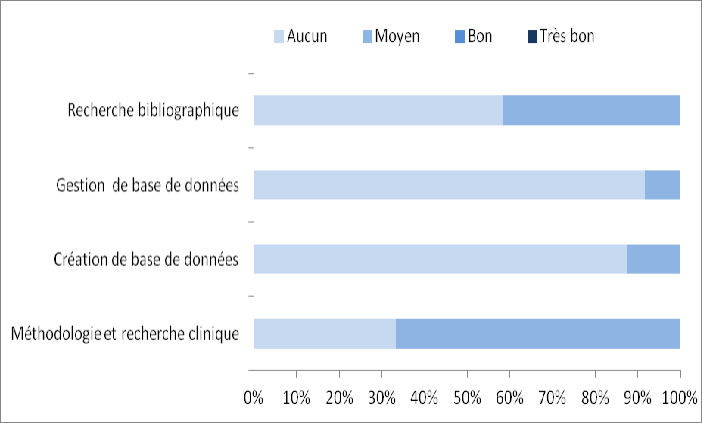
Répartition des réponses à la question «Dans les domaines suivants, quel est l'estimation de votre niveau de connaissance?»

**Attentes et connaissances initiales:** l'objectif principal des inscrits a la formation était: l'acquisition des moyens nécessaires pour réaliser et rédiger une thèse ou un article. Sur les 24 personnes ayant assisté à la première journée de formation, 14 (soit 58,2%) ne savaient pas que la formation se déroulerait en 2 phases et 23 (soit 95,8%) prévoyaient d'assister à la deuxième partie. Globalement, les étudiants étaient vierges de toute formation en création et gestion de base de données ainsi qu'en recherche bibliographique. En méthodologie de recherche clinique un quart des participants avait déjà eu des formations (Tableau 2). Les étudiants estimaient leur niveau de connaissances comme moyen à 67% en « méthodologie et recherche clinique » et 42% pour « la Recherche bibliographique » alors que pour les deux autres domaines plus de 87% estimaient avoir un niveau insuffisant (Figure 1) Concernant la finalité de leurs travaux de recherches, 23 étudiants sur 24 ont donné une réponse. Un participant voulait rédiger un article (soit 4%), 2 un mémoire (soit 9%) et 20 une thèse (soit 87%). Parmi les 24 personnes présentes la première journée, 21 (soit 88%) envisageaient de réaliser une étude et seulement 15 avaient une idée sur le type d'étude qu'ils allaient mener. Il s'agissait d'une: -étude descriptive pour huit personnes (soit 53%), -étude transversale pour trois personnes (soit 20%), -étude analytique pour trois personnes (soit 20%) -série de cas pour un participant (soit 7%).

**Tableau 2:** Répartition des réponses à la question « Avez-vous déjà suivi une formation en… »

	Oui: n (%)	Non: n (%)
Méthodologie et recherche clinique	6(25)	18(75)
Création de base de données	1(4)	23(96)
Gestion de base de données	1(4)	23(96)
Recherche bibliographique	2(8)	22(92)

**Résultat du questionnaire distribué à la fin de chaque journée de formation. Première journée**: la totalité des personnes présentes à cette journée était persuadée que cette formation aurait un impact positif sur leurs travaux (12% « un peu », 42% « assez » et 46% « beaucoup »). Plus de 78% ont estimé qu'elle avait permis d'améliorer leurs connaissances dans les différents thèmes traités. Cette amélioration était plus importante pour la partie questionnaire (le contenu et la forme) avec des valeurs comprises entre 63 et 71% de « beaucoup ». L'appréciation était plus modérée sur la partie méthodologie de recherche clinique, où le pourcentage des personnes qui n'ont ressenti aucune amélioration atteignait 21% sur « le choix d'une question de recherche ».

**Les points forts de cette journée de formation:** pour la première journée, 23 participants (soit 96%) ont noté des points forts, parmi eux: huit personnes (soit 35%) ont trouvé le cours assez clair •neuf personnes (soit 39%) ont trouvé le cours précis cinq personnes (soit 22%) ont trouvé le cours pratique et interactif huit personnes (soit 35%) ont trouvé que le cours leur a apporté de nouvelles connaissances sur la démarche à suivre pour un travail de recherche trois personnes (soit 13%) ont trouvé que le cours permettait de comprendre ce qu'on attend d'un travail de thèse huit personnes (soit 35%) ont apprécié la partie rédaction du questionnaire et recueil de l'information, trois personnes (soit 13%) ont apprécié que le cours ait été illustré avec des exemples multiples.

**Les points faibles de cette journée de formation:** sur les 24 participants, 16 (soit 67%) ont soulevé des points faibles, parmi eux: cinq personnes ont (soit 31%) trouvé le cours long, six personnes (soit 37%) l'ont trouvé dense avec beaucoup de théorie, pour 1 personne (soit 6%), il n'y avait pas assez d'exemples concrets utilisant les erreurs faites sur d'autres thèses, une personne (soit 6%) aurait préféré que la formation soit en une seule fois au lieu de la répartir sur deux temps, cinq personnes (soit 31%) ont trouvé que la formation aurait été plus bénéfique s'ils avaient un sujet et un directeur de thèse

**Commentaire et suggestions:** seulement six participants (soit 25%) avaient des commentaires ou des suggestions, trois personnes (soit 50%) ont trouvé le cours très bien fait, deux personnes (soit 33%) ont suggéré qu'on évoque dans le cours le traitement des réponses du questionnaire sur le plan statistique, une personne a demandé qu'on mette à sa disposition la liste et les contacts des personnes susceptibles d'aider en méthodologie et analyse statistique.

**Résultats de la deuxième journée:** la totalité des personnes présentes à cette journée ont affirmé que cette formation allait leur permettre de bien mener leur travail de recherche (23% « un peu », 27% « assez » et 50% « beaucoup »). Elles ont estimé qu'elle leur avait permis d'améliorer leurs connaissances surtout pour la partie création de masque de saisie et la saisie des données

**Les points forts de cette journée de formation.** Sur les 22 participants de la deuxième journée 21 (soit 96%) ont noté des points forts: pour six personnes (soit 29%) le cours a été clair, pour cinq personnes (soit 24%) le cours leur a permis la découverte et la familiarisation avec Epi info, neuf personnes (soit 42%) ont apprécié le passage de la théorie à la pratique, deux personnes ont apprécié le fait que le support power point leur a été remis.

**Les points faibles de cette journée de formation:** parmi les participants 11 (soit 50%) ont soulevé des points faibles huit personnes (soit 73%) ont trouvé le cours long et dense. trois personnes (soit 27%) ont soulevé des problèmes d'ordre technique à savoir le logiciel qui bugue et qui est incompatible avec le Mac, pour quatre personnes (soit 36%) le cours aurait été beaucoup plus bénéfique s'ils avaient un sujet de thèse.

**Commentaires et suggestions:** seulement deux participants sur 22 (soit 9%) ont eu des commentaires ou suggestions. Il s'agissait de deux personnes ayant demandé plus de pratique avec exercices.

**Résultats de la troisième journée:** la totalité des personnes présentes à cette journée était persuadée que l'impact de cette formation sur leurs travaux sera « assez 68% » voire « très important 31%» et plus de 95% ont estimé qu'elle a permis d'améliorer leurs connaissances dans les différents thèmes traités.

**Les points forts de cette journée de formation:** sur les 22 participants de la troisième journée 18 (soit 82%) ont noté des points forts: pour quatre personnes (soit 22%) le cours a constitué une véritable aide en pré thèse; trois personnes (soit 17%) ont dit que le cours leur a apporté des connaissances utiles, trois personnes (soit 17%) ont trouvé le cours clair; six personnes (soit 33%) ont trouvé le cours pratique et interactif; pour cinq personnes (soit 28%), le cours a permis d'apprendre à utiliser PubMed; pour une personne (soit 6%), le cours a permis d'apprendre à utiliser le Mesh; pour deux personnes (soit 11%), le cours a permis de se doter d'un arsenal assez important de sites de recherche internet pour faire sa recherche bibliographique.

**Les points faibles de cette journée de formation:** parmi les participants 21 (soit 96%) ont soulevés des points faibles: pour sept personnes (soit 33%), le cours était assez dense; ceux qui estimaient n'avoir aucun niveau dans les quatre domaines avant la formation et ceux qui estimaient avoir un niveau moyen n'a pas objectivé de différence statistiquement significative, sauf pour le thème « méthodologie de recherche »; pour quatre personnes (soit 19%), le cours était long; deux personnes (soit 10%) ont trouvé que l'effectif était trop important; pour qu'un seul formateur puisse gérer l'ensemble des étudiants pour quatre personnes (soit 19%), la durée consacrée au logiciel End Note© était trop courte; pour trois personnes (soit 14%) la manipulation était difficile; six personnes (soit 29%) ont soulevé des problèmes de compatibilité avec Mac et de connexion Internet.

**Commentaire et suggestions:** sur 22 participants seulement trois (soit 14%) ont eu des commentaires ou suggestions: une personne (soit 33%) a suggéré la manipulation directe sur écran pour éviter les déplacements et la répétition des explications par l'animateur; deux personnes (soit 67%) ont demandé qu'on leur envoie des cours par email; deux personnes (soit 67%) ont suggéré l'utilisation d'autres logiciels gratuits et plus pratique que End Note^®^, par exemple Zotero^®^.

**Résultat du questionnaire distribué à la fin de la formation: n=22.** Cette partie de l'évaluation a concerné trois aspects: le contenu, le déroulement et les présentations.

**Contenu:** concernant les différents aspects du contenu (la clarté des objectifs, la richesse du contenu, l'adaptation aux connaissances déjà acquises, la clarté des consignes au cours des TD, l'apport par rapport à leur sujet de recherche et l'acquisition de nouvelles connaissances) plus de 75% des étudiants ont été plutôt satisfaits. Ils ont plus coté «l'acquisition de nouvelles connaissances »

**Déroulement:** sur le déroulement des cours, mis à part le rythme que plus de 50% ont trouvé inadapté au leur, plus de 80% les participants ont été satisfaits pour les autres aspects évalués à savoir: l'interactivité, le matériel utilisé et l'adéquation entre cours théoriques et exercices des TD.

**Présentation.** Côté présentation plus de 95% ont été satisfaits des différents aspects évalués: clarté, support de cours, notion à assimiler et fil conducteur.

**Jugement global des acquis:** sur les quatre thèmes traités au cours de la formation (« création de base de données », « gestion de base de données », « recherche bibliographique » et « méthodologie de recherche clinique ») 85% des participants ont considéré que leurs connaissances étaient améliorées. Davantage pour la recherche bibliographique où le pourcentage atteignait les 100%. Nous avons comparé ce jugement en attribuant un score de 0 à 3 aux réponses des participants, entre ceux qui ont déjà suivi une formation dans ces domaines et ceux qui ne l'ont pas fait. Aucune différence statistiquement significative n'a été objectivée. L'ensemble des étudiants a estimé que cette formation a permis d'améliorer leurs connaissances. De même, la comparaison entre le groupe qui estimait avoir un niveau moyen et celui qui estimait n'avoir aucun niveau en méthodologie était en faveur du premier groupe (p=0,03) (Tableau 3). Enfin, pour 77% des participants, la formation leur a permis d'acquérir une autonomie dans leurs travaux de recherche.

**Tableau 3:** Comparaison du jugement des participants sur les quatre thèmes traités aux cours de la formation entre ceux ayant estimé n’avoir aucun niveau avant (groupe A) et ceux ayant ont estimé avoir un niveau moyen (groupe B)

Formation suivie	Groupe A Moyenne +/- écart type (effectif=n)	Groupe B Moyenne +/- écart type (effectif=n)	P
La méthodologie	1,71/0,95(7)	2,54/0,66(13)	0,03^+^
La création de base de données	2,41/0,61(17)	2,33/0,57(3)	0,84
La gestion de base de données	2,22/0,64(18)	1,50/0,70(2)	0,15
La recherche bibliographique	2,54/0,51(13)	2,43/0,53(7)	0,66

**Les points forts de la formation:** sur les 22 participants qui ont répondu au questionnaire final, 19 (soit 86%) ont noté des points forts répartis comme suit: sept personnes (soit 37%) ont trouvé qu'en totalité la formation était riche, très intéressante surtout en pré thèse, six personnes (soit 32%) ont trouvé que la formation leur a apporté de nouvelles connaissances. six participants (soit 32%) ont apprécié le coté pratique de la formation et spécialement l'élaboration du questionnaire, sept personnes (soit 37%) ont aimé la partie: création de masque de saisie avec Epi Info. 10 personnes (soit 53%) ont trouvé que la partie: « recherche bibliographique en utilisant Pub Med » était très utile.

**Les points faibles de cette formation:** les participants qui ont soulevé des points faibles étaient au nombre de 16 (soit 73%). Ils ont soulevé 3 types de problèmes:

**Technique:** problème de compatibilité des logiciels avec le Mac et problème de connexion Wifi (cinq personnes: soit 31%),

**Organisationnel:** la formation était longue et dense (six personnes: soit 37%) -le rythme de la formation était accéléré (trois personnes: soit 19%) -le temps accordé a End Note était très court (quatre personnes: soit 25%), -la répartition de la formation en deux temps avec un intervalle assez important (une personne: soit 6%) -la diffusion de l'information sur la formation qui était restreinte (1 personne: soit 6%)

**Logistique:** l'effectif très important par rapport à la salle de cours (2 personnes) -Les repas étaient payants (1 personne).

**Commentaires et suggestions:** sur cette partie du questionnaire 5 participants (soit 23%) ont répondu, ainsi: trois participants (soit 60%) ont trouvé la formation bonne et ont remercié les animateurs; un participant (soit 20%) a suggéré d'organiser une autre séance 2 mois plus tard pour renforcer les connaissances retenues; une personne (soit 20%) a demandé de leur fournir un mode d'emploi simplifié pour les logiciels utilisés dans la formation.

## Discussion

**Méthode:** La méthode que nous avons utilisée pour évaluer notre formation à savoir « l'EEE » est une méthode controversée [15]. Pour les enseignants, c'est un moyen de les noter et de les critiquer. Ils voient en elle un moyen donné aux étudiants de les juger. Ces derniers ont été longtemps considérés comme « un acteur passif du processus de l'enseignement ». Les experts en évaluation pédagogique ont un autre avis. Pour eux, « l'EEE » concerne l'enseignement dans ses différents aspects et non pas l'enseignant. C'est un moyen de faire participer activement les apprenants dans l'amélioration du processus de transfert des connaissances. [16, 17] Les questionnaires habituellement utilisés dans ce genre d'évaluation sont polymorphes et non standardisés. Les enseignants reprochent à ces questionnaires la présence de nombreuses questions à réponses ouvertes, donc subjectives. Cette critique se dissipe au vu de la richesse et de l'importance des résultats obtenus [18]. Cela dit, le questionnaire que nous avons utilisé peut être modifié et amélioré pour les évaluations ultérieures.

**Résultats:** Les enseignants de notre formation, étant conscients des points positifs de cette méthode d'évaluation, ont contribué à sa mise en œuvre du départ jusqu'à la fin. Ainsi leur avis a été pris en considération depuis l'élaboration du protocole et des questionnaires de l'enquête jusqu´à la discussion des résultats. Le taux de réponse aux différents questionnaires que nous avons distribué était de 100%, ce qui peut être expliqué par la conviction des participants de l'intérêt de leur participation et leur implication dans ce type d'évaluation. La proximité des deux HIA Sainte Anne (Toulon) et Laveran (Marseille) du CESPA expliquait la forte présence de leurs internes (92% des participants), mais avoir 2 internes de Paris et 2 nutritionnistes témoignait du début d'une prise de conscience d'envergure pour la formation. Une formation répond habituellement à un besoin. Ce besoin peut être exprimé par les apprenants qui ressentent un manque dans un ou plusieurs domaines pour accomplir des taches demandées. Il peut être exprimé par des enseignants qui jugent que leurs étudiants ont des lacunes à combler pour réaliser certains travaux; ou par l'institution qui estime que face à une nouvelle situation les étudiants ont besoin d'une formation complémentaire. Dans notre formation nous avons eu les trois cas de figure: l'institution représentée par la faculté de médecine et le Service de santé des armées qui voulaient mettre à niveau les travaux de thèse des internes; les enseignants qui ont constaté au cours de leurs encadrements de thèse que les internes avaient besoin d'une formation; les étudiants qui, à travers les résultats de l'évaluation, avaient des attentes qui rejoignaient les objectifs de la formation. Ce besoin ressenti et exprimé par les internes se confirme à travers plusieurs parties de nos résultats: la plupart des participants prévoyaient d'assister à la deuxième partie de la formation, même si 58% affirmaient ne pas être au courant qu'elle allait se dérouler sur deux temps; la majorité affirmait avoir un niveau de connaissance médiocre ou aucune formation au préalable dans les domaines traités sauf peut être en méthodologie et recherche clinique.

Les attentes des participants ont été largement satisfaites le long de la formation que ce soit du point de vue de la forme ou du fond. Ainsi, à la fin de chaque journée et à la fin de la formation, les participants ont vu leurs connaissances s'améliorer et s'enrichir de nouveauté de façon significative même pour ceux qui avaient déclaré avoir des pré- requis. Ils ont estimé que cela aura nécessairement un impact positif sur leurs travaux de recherche. Le recueil des commentaires des participants, de façon ouverte, sur les points forts et les points faibles a montré que la formation était très bénéfique. Il nous a permis d'un côté d'avoir une idée détaillée sur leur vision de ce type de formation. Il nous a permis aussi de faire sortir des points à renforcer ou à améliorer en concertation avec les responsables. Lorsque nous avons demandé si le rythme de la formation a été adapté à leur rythme d'apprentissage plus de 50% ont répondu négativement. Les réponses ouvertes nous ont apporté plus de précisions sur ce point. Ainsi nous avons constaté qu'un tiers des participants se plaignaient de la densité et de la longueur de certains cours: plus spécialement celui de la méthodologie de recherche clinique et de la gestion des références bibliographiques. Pour le premier cours «méthodologie de recherche clinique », l'enseignant estime que densité et longueur sont deux choses difficiles à satisfaire en même temps. Autrement dit, étaler son cours sur une plage horaire plus longue le rend moins dense mais plus long, et alléger son contenu se fera aux dépend des messages qu'il veut faire passer. Sur ce même module, 25% des participants affirment avoir déjà suivi une formation. Dans ce cas, un recueil détaillé sur les pré-requis des participants avant la formation pourra aider à choisir les thèmes sur lesquels il faut consacrer plus de temps et les thèmes qu'il faut traiter brièvement. Pour le deuxième cours « Recherche bibliographique et gestion des références », les deux côtés, à savoir étudiants et enseignant, étaient d'accord sur le problème de rythme. Pour les solutions, et en concertation avec l'enseignant, nous proposons de répartir les étudiants sur plusieurs groupes de faible effectif et sur deux ou trois demi-journée, ce qui pourrait donner satisfaction. Ainsi les explications et les manipulations sur PC se feront plus facilement. Grâce aux questions à réponses ouvertes, nous avons pu constater les problèmes techniques tels ceux de la connexion et de compatibilité des logiciels. La mise à disposition d'une salle informatique constituerait à nos yeux une très bonne solution qui pourrait renforcer en même temps l'infrastructure du CESPA pour jouer son rôle de structure de formation.

**Limites:** l'importance des résultats que nous avons obtenus ne nous dispense pas de parler de certaines limites de notre travail. Ces limites ne sont pas en réalité liées au travail en lui-même mais plus au contexte de son déroulement et à la spécificité de la méthode utilisée. Le premier fait concernera éventuellement l'effectif faible à savoir 27 participants. Cette remarque se dissipe au vu de la spécificité de la formation où le nombre des inscrits reste réduit et au taux de réponse que nous avons pu avoir (100%). Le deuxième point concernera peut être la discussion car nous n'avons pas pu comparer nos résultats à ceux d'autres travaux. Cela est dû au fait que nous avons utilisé la méthode d'évaluation « EEE ». Les résultats de cette méthode restent spécifiques à chaque formation évaluée. Ainsi, toute comparaison serait erronée. Par contre, les résultats de notre travail sont ceux d'une première évaluation faite sur cette formation d'aide à la rédaction de la thèse. Ceux-ci pourront être comparés aux évaluations ultérieures pour la même formation.

## Conclusion

Pour nous, ce travail n'était pas seulement une évaluation d'un enseignement. C'était aussi le moyen d'installer une certaine complicité entre les étudiants et les enseignants où les deux parties sont considérées comme deux acteurs principaux pour atteindre un objectif commun: « AMELIORER ». L'instaurer pour toute formation ultérieure contribuera certainement à la réussite de la démarche qualité que le CESPA a mis en œuvre pour toutes ses activités.

### Etat des connaissances actuelles sur le sujet

- Classiquement trois sortes d'évaluations sont distinguées: l’évaluation diagnostique, l’évaluation formative et l’évaluation sommative;
- L’évaluation d’un enseignement par les étudiants (EEE) est l’un des outils de l’évaluation formative;
- Cette méthode, EEE, est connue et déjà utilisée dans le monde anglo-saxon depuis le début du XXème siècle au niveau de l’université de Harvard.

### Contribution de notre étude à la connaissance

- Promouvoir l’utilisation de l’Evaluation d’un Enseignement par les Etudiants (EEE) comme méthode d’évaluation peu fréquente dans le monde francophone.
- Faire participer les enseignants et les étudiants en même temps dans lévaluation des formations.
- Rompre les barrières qui séparent les enseignants et les étudiants et empêchent l’amélioration des formations.
